# Pose-Driven Cow Behavior Recognition in Complex Barn Environments: A Method Combining Knowledge Distillation and Deployment Optimization

**DOI:** 10.3390/ani16091301

**Published:** 2026-04-23

**Authors:** Jie Hu, Xuan Li, Ruyue Ren, Shujie Wang, Mingkai Yang, Jianing Zhao, Juan Liu, Fuzhong Li

**Affiliations:** 1 College of Software, Shanxi Agricultural University, Jinzhong 030801, China; 18735836657@163.com (X.L.); renruyue03@163.com (R.R.); shujieyx@163.com (S.W.); ymk871016wy@163.com (M.Y.); supernubyzcmv587@163.com (J.Z.); 2 Department of Basic Sciences, Shanxi Agricultural University, Jinzhong 030801, China; liujuannk@sxau.edu.cn

**Keywords:** cattle behavior recognition, keypoint detection, pose representation, knowledge distillation

## Abstract

Cattle behavior constitutes important phenotypic information reflecting animals’ health status, activity level, and welfare condition, and is therefore of considerable significance for automated monitoring and precision management in smart livestock farming. However, under complex barn conditions, cattle behavior recognition is easily affected by factors such as illumination variation, partial occlusion, background interference, and individual differences, thereby reducing recognition stability and generalization capability. To address these challenges, this study proposes a pose-driven method for cattle behavior recognition in complex barn environments. First, a 16-keypoint annotation scheme suitable for describing bovine posture, termed cow16, was constructed. Based on this scheme, OpenPose was employed to extract heatmaps (HMs) and part affinity fields (PAFs), which were then used to build an intermediate HM/PAF posture representation. Subsequently, this representation was taken as the input to a lightweight convolutional neural network for classifying three behavioral categories: stand, walk, and lying. On this basis, class-imbalance correction during training and a multi-random-seed logits ensemble strategy during inference were further introduced. In addition, knowledge distillation was adopted to transfer knowledge from a high-performance teacher model to a lightweight student model. Experimental results demonstrate that training-stage class-imbalance correction and inference-stage multi-random-seed logits ensembling exhibit strong complementarity; when combined, the AB configuration improves the test-set Macro-F1 by 3.83 percentage points. Moreover, the distilled student model still achieves competitive recognition performance while maintaining 1× inference cost, indicating a favorable trade-off between accuracy and efficiency. This study provides a useful reference for deployment-oriented cattle behavior recognition in smart farming scenarios and offers a lightweight technical basis for subsequent practical applications.

## Simple Summary

Automated monitoring of cattle behavior is essential for precision livestock farming, as it provides vital early warnings for health and welfare issues, such as lameness and estrus. However, real barn environments—characterized by poor lighting, shadows, and crowding—often limit the reliability of standard camera systems. To overcome these practical challenges, our study introduces a novel “pose-driven” approach. Instead of analyzing the entire image, our system first detects 16 key body points on a cow to build a structural “skeleton”. This enables the computer to focus strictly on the animal’s posture while ignoring messy background interference. We then trained a lightweight artificial intelligence model to classify whether the cow is standing, walking, or lying. To ensure practical farm application, we applied techniques to prevent the AI from being biased by common behaviors and compressed the software to run efficiently on low-cost hardware. Our results demonstrate that this optimized approach significantly improves recognition stability over standard baseline models under complex conditions, without increasing computational overhead. Ultimately, this lightweight and deployment-oriented framework provides a useful basis for cattle behavior monitoring under complex barn conditions.

## 1. Introduction

Cattle behavior is an important phenotypic indicator reflecting animals’ health status, activity level, and welfare condition. Routine behaviors such as standing, walking, and lying are closely associated with lameness warning, estrus monitoring, calving observation, and precision feeding management [1,2,3,4,5]. Although traditional manual observation can provide some behavioral information, it is labor-intensive, highly subjective, discontinuous, and difficult to scale, making it inadequate for the long-term, continuous, and objective monitoring required in modern smart livestock farming. In contrast, machine-vision-based behavior recognition offers the advantages of being non-contact, automated, and capable of sustained monitoring, and has therefore attracted extensive attention in precision livestock farming and smart breeding research.

Although existing studies have made progress in cattle behavior recognition, pose estimation, and temporal modeling, automatic cattle behavior recognition in real barn environments still faces several challenges. On the one hand, complex illumination, occlusion, background interference, and individual differences can undermine the stability of appearance-based recognition. On the other hand, although temporal models can improve behavior discrimination by exploiting dynamic information from consecutive frames, their computational complexity and deployment cost are relatively high, which limits their suitability for deployment-oriented use under resource-constrained scenarios. The present study does not aim to deny the value of temporal modeling; rather, it deliberately focuses on frame-level cattle behavior recognition in order to evaluate the effectiveness of pose-based intermediate representation under complex barn conditions within a lightweight and deployment-oriented framework. Therefore, there remains substantial room for further research on single-frame, lightweight, and stable cattle behavior recognition methods for complex barn environments.

To address these issues, this study develops a pose-driven framework for cattle behavior recognition in complex barn scenes. Based on a 16-keypoint cattle annotation scheme (cow16), the framework uses OpenPose to extract heatmaps (HM) and part affinity fields (PAF), constructs an HM/PAF-based intermediate pose representation, and then employs a lightweight convolutional neural network to recognize three behavior categories: stand, walk, and lying. Furthermore, the stability of the model is enhanced at both the training and inference stages, and a high-performance teacher model is compressed into a lightweight student model through knowledge distillation so as to balance recognition performance with deployment-oriented requirements. The main contributions of this study are as follows:

(1) For side-view cattle scenes in complex barns, a cattle behavior recognition framework based on the cow16 keypoint scheme and HM/PAF intermediate pose representation is constructed, shifting behavior discrimination from appearance-dominant cues toward the use of postural structural information.

(2) To improve robustness under class imbalance and prediction fluctuation, training-stage class-prior correction and inference-stage multi-seed ensembling are jointly introduced.

(3) To improve deployment efficiency, a knowledge distillation strategy is introduced to compress a high-performance ensemble teacher model into a single student network, thereby preserving most of the performance gains while maintaining low inference cost and providing a lightweight solution for subsequent cattle behavior monitoring applications.

## 2. Related Work

### 2.1. Cattle Behavior Recognition in Barn Environments

Considerable efforts have been devoted to cattle behavior recognition in barn environments including vision-based classification, spatiotemporal modeling, lightweight methods, and multi-object behavior analysis. McDonagh et al. used vision-based methods to classify dairy cow behaviors such as standing, lying, walking, feeding, and drinking [6]. Li et al. investigated early lameness detection in dairy cows based on micro-movements and spatiotemporal features [7]. Lodkaew et al. proposed CowXNet for automatic estrus detection in dairy cows [8]. Speroni et al. assessed whether dairy cows were approaching calving through posture changes [9]. Maw et al. employed an absorbing Markov chain model to predict calving time [10]. In recent years, with the advancement of deep learning models, researchers have further applied 3D convolutional networks, temporal modeling networks, and lightweight detection networks to cattle behavior recognition tasks. Wang et al. proposed the E3D network for recognizing basic locomotor behaviors in dairy cows [11]. Gao et al. developed a CNN-Bi-LSTM model for cattle behavior classification in complex environments [12]. Yao et al. proposed a lightweight behavior recognition model for real-time monitoring [13]. Wu et al. improved cattle behavior recognition through dynamic mechanisms and multi-scale feature fusion [14]. Mu et al. introduced an improved lightweight model for beef cattle behavior recognition across multiple scenarios [15]. Tong et al. further combined behavior recognition with multi-object tracking to meet the demand for continuous monitoring in complex barn environments [16]. These studies indicate that machine vision can provide effective technical support for cattle behavior monitoring, yet substantial differences remain across tasks in terms of input format, feature representation, and deployment requirements.

### 2.2. Pose Estimation and Pose-Based Cattle Analysis

In addition to direct appearance-based recognition, pose estimation provides a more interpretable and structured representation for cattle behavior recognition. Gong et al. investigated multi-cow keypoint extraction [17]. Psota et al. achieved long-term tracking of group-housed livestock by combining keypoint detection with MAP estimation [18]. Fan et al. improved cattle keypoint detection performance using a compact multi-branch network [19]. Li et al. proposed a deep cascaded convolutional model to achieve robust cattle keypoint estimation under real farm conditions [20]. Russello et al. further incorporated temporal information and proposed T-LEAP for pose estimation of walking dairy cows [21]. Li et al. achieved real-time cattle pose estimation in complex scenes based on an improved RTMPose [22]. Kim et al. applied deep-learning-based pose estimation to the analysis of head and ear posture in cattle for animal welfare research [23]. Meanwhile, OpenPose and its Part Affinity Fields method have provided an important technical foundation for keypoint association and skeleton construction in complex scenes [24], while markerless keypoint detection methods such as DeepLabCut have also advanced animal pose analysis [25].

It should be noted that the studies summarized in Table 1 differ in dataset scale, label definition, input modality, and application scenario; therefore, this comparison is intended for contextual positioning rather than a strictly controlled head-to-head benchmark.

## 3. Materials and Methods

Cattle behavior recognition in real barn environments is constrained by both complex imaging conditions and deployment-oriented requirements. The data used in this study were collected from different barns, at different time periods, under varying illumination conditions, and from different individual cattle, thereby providing relatively comprehensive coverage of real-world variations such as backlighting, shadows, reflections, occlusions, as well as differences in body size, coat color, and posture among individuals. Accordingly, the proposed model is required not only to exhibit strong environmental robustness, but also to maintain stable recognition performance across different scenes and individuals. To improve the rigor of the experimental evaluation, sample consistency and correlation were carefully considered during data organization and partitioning. High-similarity samples, such as adjacent frames, frames from the same video clip, samples collected during the same time period, or samples from the same individual animal, were avoided as much as possible from being assigned to different data subsets. In this way, the risk of data leakage was reduced and the credibility of the test results was enhanced.

**Figure 1:**
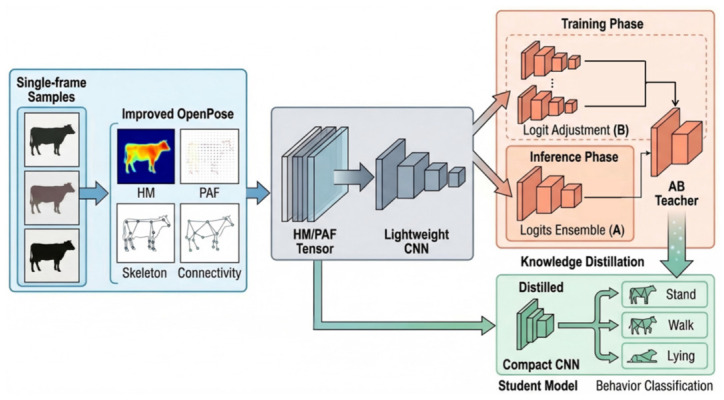
Overall pipeline of the pose-driven cattle behavior recognition framework.

The methodological design of this study was motivated by two practical considerations in complex barn environments: robustness to visual interference and suitability for lightweight use under resource-constrained conditions. First, direct appearance-based classification is easily affected by illumination variation, background clutter, partial occlusion, and inter-individual differences. For this reason, OpenPose (version 1.7.0) was introduced to convert raw cattle images into an HM/PAF-based intermediate pose representation, so that the downstream classifier could focus more on structural posture cues than on unstable appearance texture. Second, because the present task involves limited and imbalanced data, the framework was further designed to address both class-prior bias and prediction variance. Logit adjustment was therefore adopted during training to reduce the effect of class imbalance, whereas multi-random-seed logits ensembling was introduced during inference to improve prediction stability. Finally, since multi-model ensembling increases inference cost, knowledge distillation was incorporated to transfer the teacher model’s discriminative capability to a single lightweight student network. In this way, the overall framework was designed not as a simple combination of independent techniques, but as a task-oriented pipeline for frame-level cattle behavior recognition that jointly considers robustness, stability, and deployment efficiency. The overall workflow is illustrated in Figure 1.

### 3.1. Network Architecture

The proposed method adopts an overall two-stage network architecture, consisting of a pose intermediate representation generation stage and a behavior classification stage. In the first stage, a single-frame cattle image is taken as input, and OpenPose based on the cow16 scheme is used to extract keypoint heatmaps (HM) and part affinity fields (PAF). These two components are then concatenated along the channel dimension to form a fixed-size HM/PAF pose tensor. This representation not only preserves the response distribution of keypoints and the connection relationships among body parts, but also alleviates, to a certain extent, the influence of background noise, illumination variation, and partial occlusion on the downstream classification task. In this way, the behavior recognition problem is transformed from an appearance-driven task into a pose-driven one.

In the second stage, a lightweight convolutional neural network is employed to extract features from the HM/PAF pose tensor and classify cattle behaviors into three categories: stand, walk, and lying. Specifically, the second-stage classifier adopts a lightweight convolutional architecture consisting of three convolutional blocks with 64, 128, and 256 output channels, respectively. After global average pooling, the extracted features are passed through a two-layer fully connected head to produce the final predictions for the three behavior categories. To further enhance the robustness of the model in real barn environments, logit adjustment is introduced during training for class-imbalance correction, while a multi-random-seed logits ensemble strategy is adopted during inference to reduce prediction variance. On this basis, an AB Teacher is constructed. Finally, through knowledge distillation, the ensemble teacher model is compressed into a single student network, thereby reducing deployment cost while maintaining strong recognition performance.

In summary, the overall network architecture of this study establishes a complete technical pipeline of pose representation construction–enhanced behavior classification–distillation-based compression and deployment.

### 3.2. Pose Representation Construction

**Figure 2:**
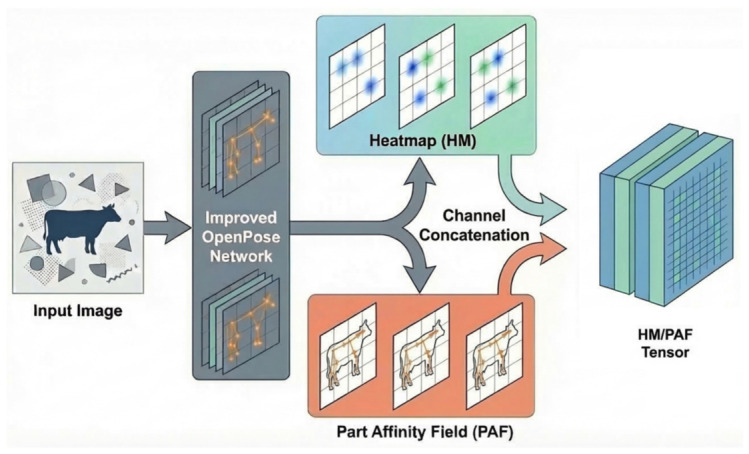
Schematic illustration of the construction of the HM/PAF intermediate pose representation.

To reduce the interference of background texture, illumination variation, and partial occlusion on behavior recognition in complex barn environments, this study constructs an HM/PAF-based intermediate pose representation by using OpenPose to extract keypoint heatmaps (HM) and part affinity fields (PAF) from single-frame images based on the cow16 keypoint scheme. In the present setting, cow16 consists of 16 keypoints and 15 connection edges; accordingly, a total of 16 heatmap channels and 30 PAF channels are generated. After concatenation, an HM/PAF tensor with 46 channels is obtained and used as the input to the subsequent classification network, as shown in Figure 2.

Because the original OpenPose framework was primarily designed for human pose estimation, its keypoint definitions are not consistent with the structural characteristics of the bovine head, trunk, and limbs, making it unsuitable for direct use in cattle behavior representation. To meet the requirements of this task, the keypoint system was redefined specifically for cattle, and a 16-keypoint annotation scheme (cow16) suitable for bovine posture description was established. On this basis, keypoint heatmaps (HM) and part affinity fields (PAF) were generated. Specifically, an HM is used to describe the response intensity and confidence distribution of each keypoint in two-dimensional space, whereas a PAF is used to characterize the directional relationships and connection strengths between adjacent keypoints, thereby representing the topological structure of the bovine skeleton. This design enables the pose representation to more accurately reflect the skeletal topology and the motion states of key body parts, thus providing structured input for subsequent behavior classification.

Based on this representation, HMs and PAFs are concatenated along the channel dimension to construct a fixed-size HM/PAF tensor, which is then fed into the subsequent lightweight convolutional neural network for behavior classification. Compared with directly using discrete keypoint coordinates, the HM/PAF representation preserves both the confidence distribution of keypoints and the connectivity relationships among body parts, thereby providing a more complete expression of the spatial contextual information of bovine posture. This representation transforms the behavior recognition task from one that relies primarily on appearance texture into a classification problem based on pose structure. As a result, it can, to some extent, mitigate the interference caused by complex imaging conditions such as backlighting, reflections, shadows, and occlusion, and provide a more stable and interpretable feature basis for discriminating among the three behavior categories of stand, walk, and lying.

The OpenPose-based pose estimator was adapted to the cow16 keypoint scheme and initialized from ImageNet-pretrained VGG19-BN weights. A total of 2429 training samples, 324 validation samples, and 324 test samples were used for pose estimation. During training, keypoint-guided cropping was first applied, followed by square letterbox resizing with an input size of 368. The optimizer was AdamW, with an initial learning rate of 1 × 10^−4^, a batch size of 8, and 60 training epochs. The best checkpoint on the validation set was then used to generate the HM/PAF representation for the downstream behavior classification stage.

### 3.3. Training-Time Logit Debiasing

In real barn environments, cattle behavior data usually exhibit class-imbalance issues, as different behaviors vary in both occurrence frequency and duration. This can easily cause the model to become biased toward majority classes during training. To mitigate the prediction bias induced by imbalanced class priors, this study introduces a logit adjustment strategy during training to explicitly calibrate the classifier outputs [26].

Let the number of samples in class c in the training set be $n_{c}$, and let the total number of training samples be N. The class prior for class c is then defined as:

(1) $$\pi_{c}=\frac{n_{c}}{N},\;c\in \{1,2,\cdots ,C\}$$

where *C* denotes the total number of classes. For an input sample x, let the original logit output of the lightweight convolutional neural network for class c be denoted as *z_c_*. After logit adjustment, the corrected output is given by:

(2) $$z_{c}\prime =z_{c}+\tau \cdot \log(\pi_{c})$$

where is a scaling coefficient used to control the strength of the class-prior correction on the logits. The corrected logits are then passed through the softmax function to obtain the predicted probability for class *c*:

(3) $$p_{c}=\frac{\exp(z_{c}\prime )}{\sum_{j=1}^{C}\exp(z_{j}\prime )}$$

For a sample whose ground-truth label is *y*, the training loss can be written as:

(4) $$L_{LA}=-\log\frac{\exp\left(z_{y}+\tau \log(\pi_{y})\right)}{\sum_{j=1}^{C}\exp\left(z_{j}+\tau \log(\pi_{j})\right)}$$

In this way, class-prior information is explicitly incorporated into the training optimization process, thereby appropriately correcting the decision boundary that would otherwise be dominated by majority classes and improving the model’s balance in recognizing minority classes and boundary samples. It should be noted that the class prior $\pi_{c}$ is estimated solely from the training set; neither the validation set nor the test set is involved in prior estimation or parameter adjustment. Therefore, this strategy does not introduce any additional risk of data leakage. Moreover, because it is applied only during training, it does not increase inference cost during deployment-oriented use.

### 3.4. Inference-Time Variance Reduction

In addition to class imbalance, under limited-data conditions, differences in model initialization and randomness during training may also lead to fluctuations in the outputs of a single model. This issue is particularly pronounced for similar behaviors such as stand and walk, where unstable predictions are more likely to occur. To reduce output variance and improve inference-stage stability, this study adopts a multi-random-seed logits ensemble strategy. Specifically, while keeping the network architecture, training data split, and major training parameters unchanged, multiple lightweight convolutional neural networks are trained independently using different random seeds only. During inference, the logits produced by these models are averaged to obtain more robust predictions.

Let $z_{c}^{\left(m\right)}$ denote the logit of class *c* output by the model trained with the m-th random seed for an input sample x, where *m*∈{1,2,⋯,M}, and M denotes the number of ensemble models. The ensembled logit for class *c* is defined as:

(5) $$z_{c}^{ens}=\frac{1}{M}\sum_{m=1}^{M}z_{c}^{\left(m\right)}$$

In this study, the number of ensemble models is set to M = 5. After obtaining the ensembled logits, the predicted probability for class *c* can be expressed as:

(6) $$\begin{array}{c} p_{c}^{ens}=\frac{\exp\left(z_{c}^{ens}\right)}{\sum_{j=1}^{C}\exp\left(z_{j}^{ens}\right)} \end{array}$$

The final predicted class is determined by the maximum a posteriori probability, namely,

(7) $$\hat{y}=\arg\underset{c\in \{1,2,\cdots ,C\}}{\max}p_{c}^{ens}$$

By averaging the outputs of multiple random-seed models, this strategy effectively suppresses the incidental errors caused by initialization differences and local optima in a single model, thereby improving the discriminative stability for boundary samples. It should be noted that this method is applied only at the inference stage and does not alter the training process of any individual model. In this study, this inference enhancement strategy is denoted as Method A. It is further combined with logit adjustment at the training stage (Method B) to form the AB Teacher used in the subsequent knowledge distillation process [27].

### 3.5. Knowledge Distillation

**Figure 3:**
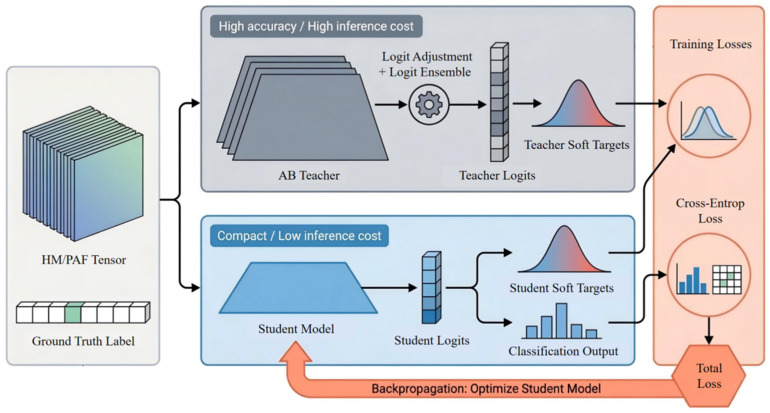
Knowledge distillation and deployment optimization based on the AB Teacher.

Although the AB Teacher, which combines logit adjustment with a multi-random-seed logits ensemble, can achieve superior recognition performance, multi-model ensembling substantially increases inference cost and is therefore less suitable for deployment-oriented use under resource-constrained conditions. To reduce model complexity while preserving recognition accuracy, this study introduces a knowledge distillation strategy. Specifically, the AB Teacher is used as the teacher model, and its discriminative capability is transferred to a single student network, thereby yielding a lightweight deployment model that balances performance and efficiency [28]. The overall knowledge distillation and deployment optimization framework based on the AB Teacher is illustrated in Figure 3.

Let the logits output by the teacher model and the student model be denoted by *z*_t_ and *z*_s_, respectively, and let T denote the temperature coefficient. The soft target distributions of the teacher and student models are then defined as:

(8) $$p_{t}^{\left(c\right)}=\frac{\exp\left(z_{t}^{\left(c\right)}/T\right)}{\sum_{j=1}^{C}\exp\left(z_{t}^{\left(j\right)}/T\right)}$$

(9) $$p_{s}^{\left(c\right)}=\frac{\exp\left(z_{s}^{\left(c\right)}/T\right)}{\sum_{j=1}^{C}\exp\left(z_{s}^{\left(j\right)}/T\right)}$$

where C denotes the total number of classes, and *p*_t_(c) and *p*_s_(c) represent the soft prediction probabilities of the teacher and student models for class c, respectively. Based on these soft target distributions, the distillation loss can be written as:

(10) $$L_{KD}=T^{2}\sum_{c=1}^{C}p_{t}^{\left(c\right)}\log\frac{p_{t}^{\left(c\right)}}{p_{s}^{\left(c\right)}}$$

Meanwhile, in order to preserve the student model’s discriminative ability with respect to the ground-truth labels, the cross-entropy loss is introduced as:

(11) $$L_{CE}=-\sum_{c=1}^{C}y_{c}{\log\hat{y}}_{c}$$

where *y*_c_ is the one-hot encoding of the ground-truth label, and ${\hat{y}}_{c}$ denotes the predicted probability of the student model for class c. The final optimization objective of the student model is therefore formulated as:

(12) $$\begin{array}{c} L=(1-\lambda )L_{CE}+\lambda L_{KD} \end{array}$$

where *λ* is the distillation loss weight used to balance hard-label supervision and soft-target regularization. In this way, the student model is able to learn the smoother and more stable class-distribution information produced by the teacher model, thereby inheriting as much as possible the recognition capability of the ensemble teacher while maintaining the inference cost of a single model [29]. Based on this strategy, this study ultimately obtains a lightweight cattle behavior recognition model suitable for deployment-oriented applications.

## 4. Results

To evaluate the performance of the proposed method for cattle behavior recognition in complex barn environments, this section first introduces the experimental setup and evaluation metrics. It then compares the overall performance of the baseline and the various improvement strategies under a unified training protocol. Furthermore, through confusion matrices, class-level metrics, deployment comparisons based on knowledge distillation, and uncertainty analysis for small-sample classes, the effectiveness of the proposed method and its deployment-oriented potential are comprehensively evaluated.

### 4.1. Experimental Setup

To ensure the reproducibility of the behavior classification experiments and the fairness of comparisons, all classification models were trained and tested under the same hardware and software environment. The detailed environment configuration and training settings for the behavior classification stage are summarized in Table 2.

It should be noted that the settings in Table 2 correspond to the downstream behavior classification stage, whereas the training details of the pose estimation stage are separately described in Section 3.2.

### 4.2. Experimental Data

The data used in this study were collected from several cattle farms in Linxian County, Shanxi Province. Raw surveillance video data were acquired under different lighting conditions, at different times of day, and from different individual cattle, thereby reflecting the complexity of cattle behavior monitoring in real farming environments. The data collection process was conducted with the informed consent of the local farm owners and adopted a non-invasive natural observation approach, ensuring the authenticity and validity of the collected data. To improve the reliability of the experiments, correlations among samples were carefully considered during data organization and partitioning, and efforts were made to avoid assigning highly similar samples—such as adjacent frames or frames from the same video clip—to different subsets.

#### 4.2.1. Data Collection

The experimental data were collected by fixed cameras installed at an oblique side-top viewing angle in the farm. Image acquisition was conducted primarily under natural lighting conditions and included real-world disturbances such as shadows, partial occlusion, and illumination changes at different times of day. The original video resolution was 2304 × 2592.

#### 4.2.2. Data Preprocessing and Partitioning

After the raw surveillance videos were collected, single-frame image samples were first extracted from the continuous video streams and organized according to the requirements of the subsequent recognition task. To ensure sample quality, invalid frames with severe blur, excessive occlusion, incomplete target regions, or keypoints that could not be reliably annotated were removed during preprocessing. The remaining images were then uniformly resized to 360 × 520 pixels to meet the input requirements of the subsequent models and to reduce additional interference caused by differences in image resolution.

**Figure 4:**
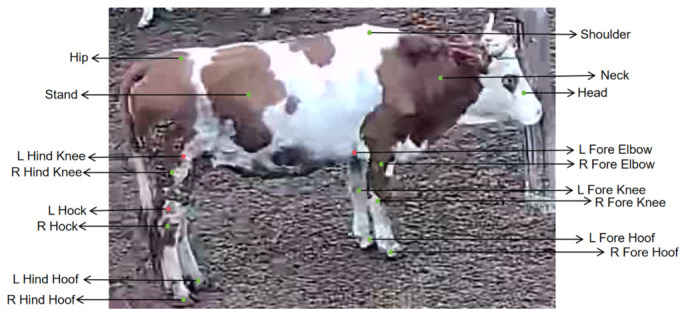
Schematic diagram of the cow16 keypoint definition.

After sample extraction, as shown in Figure 4, the Labelme tool was used to annotate 16 keypoints, and each sample was further labeled into one of three behavioral categories—stand, walk, and lying—according to the behavioral state of the cattle. The dataset was then divided into training, validation, and test sets. Ultimately, the study obtained 2413 training samples, 323 validation samples, and 323 test samples, yielding a total of 3059 valid samples, which provided the data foundation for subsequent model training and performance evaluation. The class-wise distribution of the three behavior categories across the training, validation, and test sets is summarized in Table 3. The class-wise distribution reveals a noticeable imbalance among the three behavior categories. These class counts were further used to estimate the class priors in the subsequent logit adjustment strategy.

### 4.3. Evaluation Metrics

To objectively evaluate the classification performance of the model on the cattle behavior recognition task, this study adopts Precision, Recall, and F1-score as the basic evaluation metrics, and uses Macro-F1 as the primary metric. Compared with Accuracy, which reflects only overall correctness, Macro-F1 provides a more comprehensive measure of the model’s balanced recognition performance across different behavior categories under class-imbalanced conditions, and is therefore more suitable for the three-class classification task considered in this study. For class c, Precision, Recall, and F1-score are defined as follows:

(13) $$\begin{array}{c} {Precision}_{c}=\frac{TP_{c}}{TP_{c}+FP_{c}} \end{array},$$

(14) $$\begin{array}{c} {Recall}_{c}=\frac{TP_{c}}{TP_{c}+FN_{c}}, \end{array}$$

(15) $$F1_{c}=\frac{2\cdot {Precision}_{c}\cdot {Recall}_{c}}{{Precision}_{c}+{Recall}_{c}},$$

where *TP*_c_, *FP*_c_, and *FN*_c_ denote the true positives, false positives, and false negatives for class c, respectively. On this basis, Macro-F1 is defined as the arithmetic mean of the F1-scores over all classes, namely,

(16) $$Macro-F1=\frac{1}{C}\sum_{c=1}^{C}F1_{c},$$

where C denotes the total number of classes, and in this study, C = 3. In addition, to further analyze the model’s recognition differences across categories, confusion matrices are used in subsequent analyses to examine the error distribution among the three behavior classes: stand, walk, and lying. Considering that the lying class contains relatively fewer samples, the bootstrap method is further employed to estimate its 95% confidence interval, so as to quantify the uncertainty of results for the small-sample class.

### 4.4. Overall Performance Comparison

Before comparing behavior recognition performance under different strategies, the reliability of the pose estimation stage was assessed independently, since the HM/PAF representation used by the second-stage classifier is directly derived from the predicted keypoints. On the cow16 test set, the pose estimator achieved a mean PCK@0.05 of 0.6967 and a mean PCK@0.10 of 0.8915, indicating that the generated pose representation provides sufficiently reliable structural information for downstream behavior classification.

To verify the effectiveness of the class-imbalance correction strategy at the training stage and the ensemble strategy at the inference stage, this study compared the baseline, the inference-stage ensemble strategy A, the training-stage logit adjustment strategy B, and their combination AB under a fixed data split and unified training settings. In this study, the baseline refers to a single lightweight CNN trained on the HM/PAF-based pose representation without logit adjustment, multi-seed ensemble inference, or knowledge distillation. The results of the single-model experiments are reported as the mean ± standard deviation over five random seeds, while the inference-stage ensemble results are obtained by equally averaging the logits of the five seed-trained models. The primary evaluation metric is Macro-F1 on the test set. The results are presented in Table 4.

As shown in Table 4, both the training-stage class-imbalance correction and the inference-stage multi-random-seed ensemble improve the overall model performance, with the AB combination achieving the best results. Compared with the baseline single model, the Macro-F1 of AB is improved by approximately 3.83 percentage points; compared with the single model using only strategy B, it is further improved by about 3.03 percentage points. These results indicate that the two strategies exhibit strong complementarity, and their joint use can more effectively enhance the overall performance of cattle behavior recognition in complex barn environments.

### 4.5. Comparison with External Baselines

To provide comparative context for the proposed HM/PAF-based method, three external baselines were further evaluated under the same train, validation, and test split, including two raw-image classifiers based on MobileNetV3 and ResNet-50, and a coordinate-based baseline using the cow16 keypoint coordinates and visibility indicators as input to an MLP. The comparison results are shown in Table 5.

As shown in Table 5, both raw-image baselines perform markedly worse than the pose-based methods, indicating that direct appearance-based classification is less robust under complex barn conditions. The coordinate-based MLP baseline is already competitive, suggesting that the cow16 keypoint geometry itself contains strong discriminative information. Nevertheless, the proposed HM/PAF-based framework combined with the training- and inference-stage optimization strategies still achieves the best overall performance.

### 4.6. Ablation Study on Pose Components and Ensemble Size

To further evaluate the contribution of different pose components and the effect of ensemble size, additional ablation experiments were conducted.

As shown in Table 6, the combined HM/PAF representation achieved the best performance, while HM only outperformed PAF only. This indicates that both keypoint-response information and structural relation information contribute to behavior recognition, and their combination provides a more effective pose representation.

To further examine the effect of multi-seed ensembling, the ensemble size was varied from 1 to 3 and 5 under the A and AB settings.

As shown in Table 7, ensemble size affects the two settings differently. For A, performance improves from M = 1 to M = 3, but no further gain is observed at M = 5. For AB, the performance increases consistently with larger ensemble size and reaches the best result at M = 5. Therefore, M = 5 was retained in the final AB teacher.

### 4.7. Error Analysis and Class-Level Results

**Figure 5:**
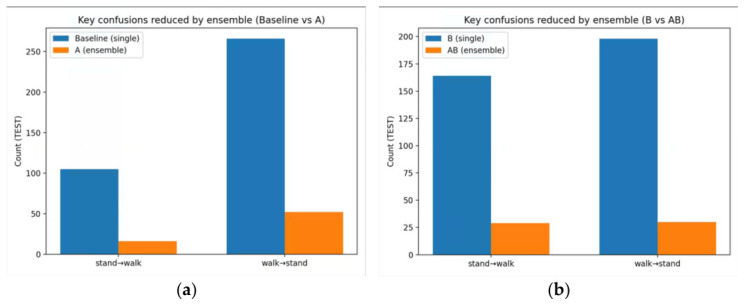
Changes in the key stand–walk confusions under different strategies. (**a**) comparison between the baseline single model and strategy A (logits ensemble). (**b**) comparison between strategy B (logit adjustment) and strategy AB (logit adjustment + logits ensemble).

To further analyze the sources of model errors, this study conducted a statistical analysis of the key confusing categories and the class-level recognition results under the best-performing configuration. The results show that the main errors of the baseline are concentrated between stand and walk, indicating that standing and walking are the most easily confused behavior categories. After introducing the inference-stage multi-random-seed logits ensemble, the key confusion between stand and walk is significantly reduced, suggesting that ensembling can effectively suppress the prediction variance caused by random initialization and improve decision stability. A further comparison between B and AB shows that logit adjustment at the training stage and ensemble inference at the inference stage have strong complementarity. While reducing the key confusion, AB also achieves the best overall performance. The changes in the key confusion between stand and walk under different strategies are shown in Figure 5.

The class-level results of the best-performing AB configuration on the test set are presented in Table 8. It can be seen that recognition performance still varies across different behavior categories. The model errors are mainly concentrated between stand and walk, whereas the lying category exhibits relatively stable discrimination performance. This indicates that the current method is capable of effectively capturing differences in bovine posture, but its ability to perform fine-grained discrimination between similar behaviors still requires further improvement.

### 4.8. Deployment Trade-Off Under Knowledge Distillation

Although the AB combination achieves the best overall performance on the test set, its inference stage requires the integration of outputs from multiple models, resulting in relatively high computational cost and making it less suitable for lightweight deployment-oriented use under resource-constrained farm conditions. To reduce inference cost while maintaining recognition accuracy, this study further introduces a knowledge distillation strategy. Specifically, the AB ensemble teacher is used as the teacher model, and its discriminative capability is transferred to a single student network, thereby yielding a lightweight recognition model that is more suitable for deployment.

The deployment comparison between the teacher model and the student model is shown in Table 9. Overall, the AB ensemble teacher achieves the highest Macro-F1 on the test set, but it also incurs the highest inference cost. In contrast, the KD-Student, while maintaining the inference cost of a single model, is still able to preserve most of the performance gains of the ensemble teacher and outperforms the corresponding single-model baseline. These results demonstrate that knowledge distillation can effectively transfer knowledge from a high-performance ensemble model to a lightweight deployment model, achieving a favorable trade-off between accuracy and efficiency in complex barn environments and providing a more feasible technical basis for subsequent deployment-oriented applications.

### 4.9. Uncertainty Analysis for the Small-Sample Class and Complexity Analysis

Because the lying category contains relatively few samples in the test set, this study further applies the bootstrap resampling method to estimate the 95% confidence intervals of Precision, Recall, and F1-score for this class, with the number of resamples set to B = 20,000, so as to quantify the uncertainty of the results under small-sample conditions. The results are shown in Table 10. Although the KD-Student achieved a slightly higher point estimate than the AB ensemble teacher for the lying category, this difference alone is insufficient to indicate that the student model performs better than the teacher model on this class. Compared with the stand–walk boundary, the lying behavior is characterized by a more stable body configuration and clearer geometric pose cues, making this category easier to preserve during distillation. At the same time, the distillation process may help the student retain the decision boundary of this relatively stable category while reducing prediction noise. In addition, because the lying class contains relatively few samples, its metric estimates are more sensitive to sample fluctuation. Therefore, the observed improvement of the student model on this category is more appropriately understood as a class-specific phenomenon under small-sample conditions rather than evidence that the student model comprehensively surpasses the teacher. This interpretation is further supported by the substantial overlap between the bootstrap confidence intervals of the two models.

In addition to recognition performance, model complexity and inference cost are also key factors in deployment-oriented applications. The results show that a single model contains approximately 0.43 M parameters, with an FP32 model size of about 1.64 MB, demonstrating favorable lightweight characteristics. Although the AB ensemble teacher achieves the best test performance, its inference cost is approximately five times that of a single model, making it less suitable for deployment-oriented use under resource-constrained conditions. In contrast, the KD-Student retains most of the performance gains of the teacher model while maintaining 1× inference cost, thereby exhibiting a better trade-off between accuracy and efficiency. Overall, the proposed method not only improves cattle behavior recognition performance in complex barn environments, but also shows good potential for deployment-oriented applications.

## 5. Discussion

The proposed pose-driven framework achieved stable cattle behavior recognition performance under complex barn conditions. The additional baseline experiments showed that pose-based representations were markedly more robust than raw-image classification, while the coordinate-based baseline was already competitive [30,31]. These results suggest that structural pose cues are more reliable than direct appearance cues for this task.

The main remaining errors were concentrated between the stand and walk categories, whereas the lying category was relatively more stable. This suggests that, under a single-frame oblique side-top view, discriminating between similar behaviors remains the primary challenge. A likely reason is that the difference between standing and walking in static images is often reflected only in subtle changes in leg position, trunk center of gravity, and local skeletal topology, which become even less distinguishable during transitional postures. Although the proposed method alleviates this type of confusion to some extent, the results still indicate that single-frame pose information alone is insufficient to fully capture the dynamic differences between similar behaviors.

From the perspective of deployment-oriented application, knowledge distillation further highlights the deployment value of the proposed method. Although the AB ensemble configuration achieved the highest recognition performance, its inference cost was relatively high. In contrast, the distilled student model retained most of the performance gains of the teacher model while maintaining the inference overhead of a single model, indicating a favorable trade-off between recognition accuracy and computational efficiency. This suggests that the proposed framework provides a lightweight technical basis for subsequent deployment-oriented applications under resource-constrained farm conditions [32].

Nevertheless, this study still has certain limitations. First, the current method is based on single-frame input and does not explicitly model temporal information; therefore, its representation of dynamic transitions between similar behaviors such as stand and walk remains insufficient. This design choice was intentional, as the current study aimed to evaluate the value of pose-based intermediate representation in a frame-level and lightweight setting rather than to optimize temporal modeling. Second, the data were mainly collected from a limited number of farms and over a limited time span, so the model’s cross-scene generalization ability still needs to be further validated using larger-scale datasets. In addition, the number of lying samples is relatively small, and the recognition results should be interpreted together with uncertainty analysis. The current study also focuses on single-target, frame-level cattle behavior recognition rather than group-housing scenarios. Future research will therefore focus on lightweight temporal modeling, multi-scene dataset expansion, and further optimization for resource-constrained deployment.

## 6. Conclusions

This study proposed a pose-driven framework for frame-level cattle behavior recognition in complex barn environments, using the cow16 keypoint scheme, HM/PAF intermediate representation, and a lightweight classification pipeline.

Experimental results showed that the proposed method achieved the best performance under the AB configuration, with a test-set Macro-F1 of 0.8125. The distilled student model still achieved 0.8006 ± 0.0083 at 1× inference cost, indicating a favorable trade-off between recognition accuracy and computational efficiency. Additional experiments further showed that the combined HM/PAF representation outperformed HM only and PAF only, while raw-image baselines performed markedly worse than the pose-based methods.

Overall, the proposed framework provides a lightweight and deployment-oriented basis for cattle behavior recognition under complex barn conditions. The current work focuses on single-target, frame-level recognition and does not yet address group-housing scenarios involving multi-object tracking, identity association, and severe mutual occlusion. Future research will further investigate temporal modeling, multi-scene dataset expansion, and lightweight optimization for edge-side applications [33,34].

## Figures and Tables

**Table 1:** Representative recent studies related to cattle behavior recognition and pose-based analysis.

Study	Input/Setting	Main Characteristic	Relation to This Study
Wang et al. (2023) [11]	Video/spatiotemporal	3D CNN-based recognition	Temporal
Gao et al. (2023) [12]	CNN + Bi-LSTM	Temporal feature fusion	Temporal
Mu et al. (2024) [15]	YOLO-based RGB input	Lightweight recognition	RGB-based
Tong et al. (2024) [16]	Recognition + tracking	Multi-object analysis	Tracking-based
This study	HM/PAF representation	Frame-level, pose-driven framework	Single-target

**Table 2:** Experimental environment settings and training parameters for the behavior classification stage.

Category	Item	Setting
Experimental environment	Operating system	Ubuntu 20.04
	CPU	Intel Xeon Platinum 8270 @ 2.70 GHz
	Memory	64 GB
	GPU	NVIDIA GeForce RTX 4090 D (24 GB memory)
	Deep learning framework	PyTorch 2.9.1
	Programming language	Python 3.10
Training parameters	Optimizer	AdamW
	Initial learning rate	0.0010
	Batch size	8
	Maximum epochs	40
	Weight decay	1 × 10^−4^

**Table 3:** Class distribution across the training, validation, and test sets.

Class	Train	Validation	Test	Total
Stand	756	105	138	999
Walk	1047	186	164	1397
Lying	610	32	21	663
Total	2413	323	323	3059

**Table 4:** Overall performance comparison of different strategy combinations on the test set.

Method	Training Module	Inference Module	Macro-F1
Baseline (single)	baseline	single	0.7742 ± 0.0034
A (ensemble)	baseline	5-seed logits ensemble	0.7876
B (logit adjustment)	Logit_adjust	single	0.7822 ± 0.0089
AB (logit adjustment + ensemble)	Logit_adjust	5-seed logits ensemble	0.8125

**Table 5:** Comparison with external baselines under the same data split.

Method	Input Representation	Module	Macro-F1
RGB baseline 1	Raw RGB image	MobileNetV3	0.5865 ± 0.0184
RGB baseline 2	Raw RGB image	ResNet-50	0.5455 ± 0.0248
Coordinate baseline	16 keypoints (x, y, v)	MLP	0.8029 ± 0.0081
Proposed baseline	HM/PAF tensor	Lightweight CNN	0.7742 ± 0.0034
Proposed best	HM/PAF tensor	AB	0.8125

**Table 6:** Ablation study on different pose components.

Method	Input Representation	Macro-F1
HM only	HM	0.7406 ± 0.0174
PAF only	PAF	0.7141 ± 0.0118
HM + PAF	HM/PAF	0.7742 ± 0.0034

**Table 7:** Effect of ensemble size on test-set Macro-F1.

Group	M = 1	M = 3	M = 5
A (baseline + ensemble)	0.7524	0.7900	0.7876
AB (logit adjustment + ensemble)	0.7674	0.7937	0.8125

**Table 8:** Class-wise recognition results of the best AB configuration on the test set.

Class	Precision	Recall	F1
Cow_stand	0.7714	0.7770	0.7742
Cow_walk	0.8210	0.8061	0.8135
Cow_lying	0.8095	0.8947	0.8500

**Table 9:** Deployment-oriented comparison between the teacher and student models.

Method	Model Type	Inference Cost	Macro-F1
B single	Single model	1×	0.7822 ± 0.0089
AB ensemble teacher	Ensemble teacher	5×	0.8125
KD-Student	Distilled single model	1×	0.8006 ± 0.0083

**Table 10:** Bootstrap 95% confidence intervals of the cow_lying class on the test set.

Setting	Precision 95% CI	Recall 95% CI	F1 95% CI
AB ensemble teacher	0.8095 [0.7250,0.9583]	0.8947 [0.7368,1.00000]	0.8500 [0.7097,0.9545]
KD-Student	0.8947 [0.7368,1.0000]	0.8947 [0.7368,1.00000]	0.8947 [0.7692,0.9787]

## Data Availability

The dataset was developed by our research team and will be made publicly accessible upon reasonable request.
